# Pertactin-Negative and Filamentous Hemagglutinin-Negative *Bordetella pertussis*, Australia, 2013–2017

**DOI:** 10.3201/eid2506.180240

**Published:** 2019-06

**Authors:** Zheng Xu, Sophie Octavia, Laurence Don Wai Luu, Michael Payne, Verlaine Timms, Chin Yen Tay, Anthony D. Keil, Vitali Sintchenko, Nicole Guiso, Ruiting Lan

**Affiliations:** University of New South Wales, Sydney, New South Wales, Australia (Z. Xu, S. Octavia, L.D.W. Luu, M. Payne, R. Lan);; New South Wales Health Pathology and Westmead Hospital, Sydney (V. Timms, V. Sintchenko);; The University of Sydney, Sydney (V. Timms, V. Sintchenko);; University of Western Australia, Perth, Western Australia, Australia (C.Y. Tay);; Perth Children’s Hospital, Perth (A.D. Keil); Institut Pasteur, Paris, France (N. Guiso)

**Keywords:** Bordetella pertussis, Pertactin, Prn-negative, filamentous haemagglutinin, Fha-negative, fim2, ptxP3, Australia, bacteria, acellular vaccine, pertussis

## Abstract

During the 2008–2012 pertussis epidemic in Australia, pertactin (Prn)–negative *Bordetella pertussis* emerged. We analyzed 78 isolates from the 2013–2017 epidemic and documented continued expansion of Prn-negative *ptxP3 B. pertussis* strains. We also detected a filamentous hemagglutinin-negative and Prn-negative *B. pertussis* isolate.

Despite high vaccination coverage, pertussis remains a major public health concern. In many industrialized countries, including Australia, whole-cell vaccine was replaced by the less reactogenic acellular vaccine (ACV). In Australia, the 3-component ACV (containing pertactin [Prn], pertussis toxin [Ptx], and filamentous hemagglutinin [Fha]) has been more widely used than the 5-component ACV (which also contains fimbrial antigen: Fim2 and Fim3).

Since 1991, when notifications began, pertussis has reemerged in Australia, and epidemics occur every 3–5 years. The largest epidemic occurred in 2008–2012; 39,000 cases were recorded at its peak in 2011 (*1*,*2*). Most *Bordetella pertussis* isolates from that epidemic belonged to 1 genetic group, referred to as single-nucleotide polymorphism (SNP) cluster I (*1**–**3*). SNP cluster I had *prn2* allele of the *prn* gene and *ptxP3* allele of the Ptx promoter (*3*).

In a study of the 2008–2012 epidemic, Lam et al. (*1*) reported a rapid increase in the number of isolates not expressing the ACV antigen Prn (Prn-negative), from 5.13% in 2008 to 77.78% in 2012. Sequencing of 22 isolates revealed 5 epidemic lineages (ELs) (EL1–EL5) and independent origins of Prn-negative strains in different ELs (*4*). A smaller epidemic occurred during 2013–2017, peaking at 22,000 cases in 2015 (Figure 1, panel A). We investigated the genotypic and phenotypic characteristics of 78 *B. pertussis* isolates from 2013–2017 to determine the epidemic trends of pertussis in Australia.

**Figure 1:**
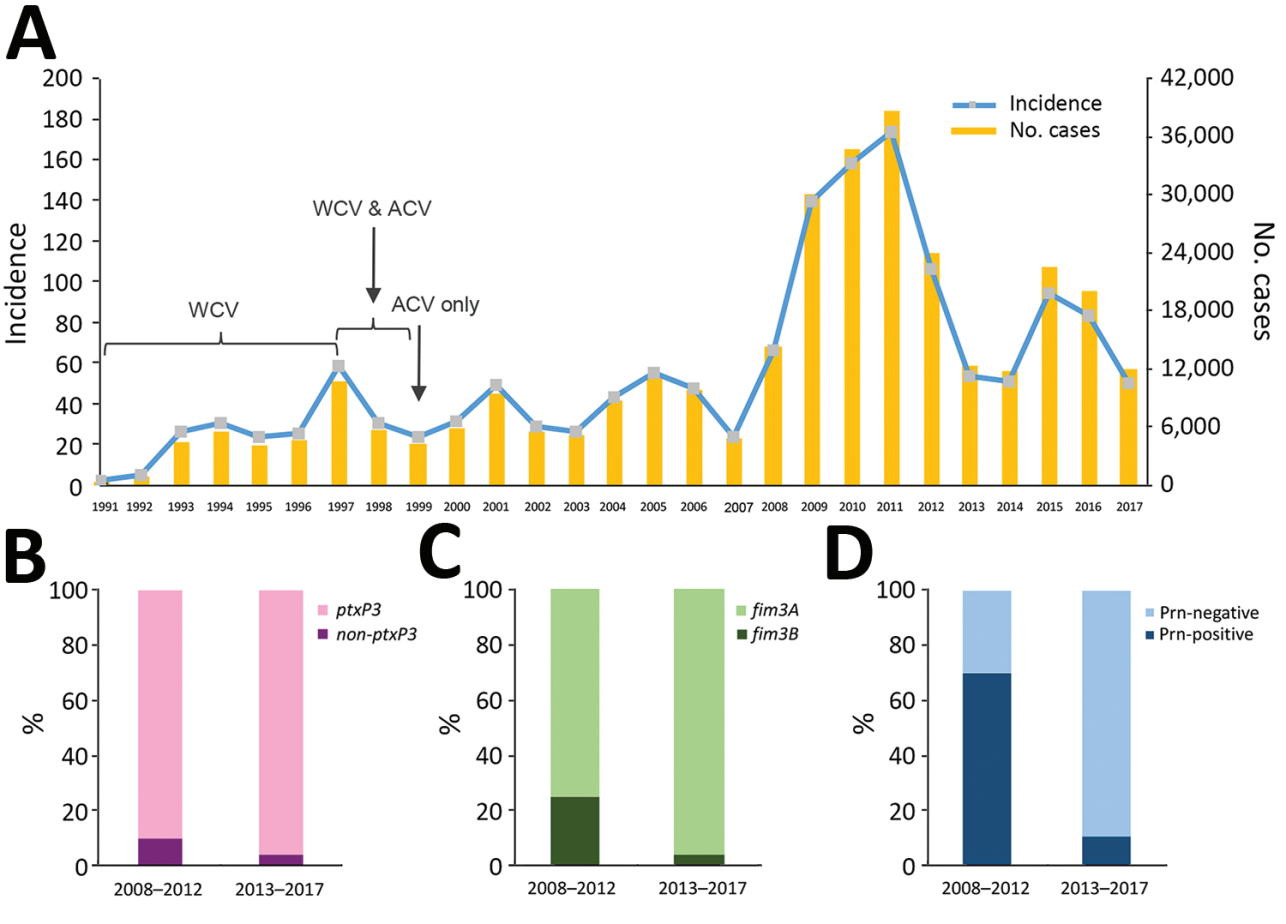
Pertussis trends and temporal changes of allele frequencies of vaccine antigens genes, Australia. A) Pertussis notifications in Australia, 1991–2017. Source: National Notifiable Diseases Surveillance System (http://www9.health.gov.au/cda/source/cda-index.cfm). Incidence is number of cases per 100,000 population. Reporting system was established in 1991. ACV was first introduced in Australia in 1997, and WCV was replaced with ACV in 1999. A large pertussis epidemic occurred during 2008–2012, and another epidemic occurred during 2013–2017. B) Percentage of *Bordetella pertussis* isolates carrying *ptxP3* allele in the 2 epidemic periods. C) Percentage of *B. pertussis* isolates carrying *fim3A* and *fim3B* allele in the 2 epidemic periods. D) Percentage of pertactin-expressing (Prn-positive) and pertactin-deficient (Prn-negative) *B. pertussis* isolates in the 2 epidemic periods. ACV, acellular vaccine; Prn, pertactin; WCV, whole-cell vaccine.

## The Study

We sequenced 78 *B. pertussis* isolates (Appendix 1 Table 1) from New South Wales (NSW) (17/78 [21.8%]) and Western Australia (WA) (61/78 [78.2%]) that were collected during the 2013–2017 epidemic. We conducted SNP detection (Appendix 1 Table 2) and examined variation in ACV antigen genes (*prn*, *ptxA*, *ptxP*, and the 2 fimbrial genes *fim2* and *fim3*). Using the SNP-based classification scheme by Octavia et al. (*3*), we typed the 78 isolates into 2 SNP profiles (SPs): SP13 (SNP cluster I, *ptxP3*, 75/78 [96.2%]) and SP18 (noncluster I, *ptxP1*, 3/78 [3.8%]). All isolates harbored the *ptxA1* allele. Most (75/78 [96.2%]) of the SP13 isolates had the *prn2* and *fim3A* alleles. The 3 noncluster I SP18 isolates had a fim3A* allele that differs from *fim3A* by a synonymous mutation (*3*) with genotype ptxP1-fim3A*-prn1. The frequency of *ptxP3* and *fim3A* alleles was higher than during the 2008–2012 epidemic (Figure 1, panels B, C). All but 1 isolate carried the *fim2–1* allele. One isolate (L2263 [SP18]) contained a *fim2* allele with a new 3-nucleotide insertion (AGA) at position 506, resulting in the insertion of a lysine in the epitope (F2.9) region of Fim2 (*5*). PROVEAN analysis (*6*) suggests that the insertion does not affect protein structure and thus might or might not affect immune recognition. We designated this allele as *fim2–3* (GenBank accession no. MG824989). Western immunoblotting showed that all isolates expressed Ptx, and all but 1 (L2228) expressed Fha. For Prn, 89.7% (70/78) isolates were Prn-negative (Figure 1, panel D), suggesting continued expansion of Prn-negative strains.

We found multiple mechanisms of *prn* inactivation in the isolates, all but 1 of which were reported previously (*1*,*7**–**9*). For most (66/70) isolates, inactivation was caused by insertion sequences (IS), including 45 IS*481*F insertions (F/R denotes insertion orientation relative to *prn*), 17 IS*481*R insertions, and 4 IS*1002*R insertions (Table). We found an IS*481*F insertion, which has been reported in *prn1* and *prn2* isolates only (*1*,*8*), in 3 of the *prn3* isolates. One Prn-negative isolate contained a SNP (C→T) in position 223, resulting in a stop codon, a mutation found previously in US isolates only (*10*). Two isolates had a deletion (position −297, 1325 [relative to the initiation codon ATG]) between the promoter and 5′ end of *prn* that was replaced with a fragment of IS*1663*, which might have mediated the deletion (Table). A similar but slightly different deletion (position −292, 1340) was reported in US isolates (*7*). We identified a new inactivation by a 4-bp deletion, from position 2020 to 2023 in *prn*, in 1 isolate (L2210) (Appendix 1 Table 1). 

**Table:** Mechanisms of pertactin deficiency and characteristics of *Bordetella* *pertussis* isolates from pertussis epidemics, Australia, 2013–2017*

Prn deficiency mechanism	Position in *prn*†	*prn* allele type	State (no. of isolates)	Year (no. isolates)	References
IS*481*F	1613	*prn2*	Western Australia (32)	2013 (13)	(*1*)
			New South Wales (10)	2014 (5)	
				2015 (11)	
				2016 (9)	
				2017 (4)	
IS*481*R	1613	*prn2*	Western Australia (12)	2013 (6)	(*1*)
			New South Wales (5)	2014 (5)	
				2015 (4)	
IS*481*F	1598	*prn3*	Western Australia (3)	2013 (1)	This study
				2014 (2)	
IS*1002*R	1613	*prn2*	Western Australia (4)	2013 (2)	(*1*)
				2016 (1)	
				2017 (1)	
Deletion	−297 to 1325	Not determined‡	Western Australia (2)	2014 (1)	(*8*), newly found in Australia
				2015 (1)	
Stop codon	223	*prn2*	Western Australia (1)	2014 (1)	(*10*), newly found in Australia
Deletion	2020–2023	*prn2*	Western Australia (1)	2013 (1)	This study

*F/R denotes IS insertion orientation relative to prn. F, forward; IS, insertion sequence; Prn, pertactin; R, reverse. †The nucleotide positions are relative to the initiation codon (ATG) of the *prn* in Tohama I. ‡*prn* allele type was not determinable because the repeat regions that define *prn* allele type was deleted in this mechanism.

One Prn-negative isolate (L2228) was also Fha-negative (i.e., Prn−, Fha−) by Western immunoblotting. The Fha inactivation probably resulted from changes within the homopolymeric G tract (site: 1078–1087) from 10 Gs to 11 Gs in *fhaB*, resulting in a downstream stop codon that produces a truncated FhaB protein (*11*). Both Illumina and Sanger sequencing (Appendix 2) showed a mixture of 10 Gs and 11 Gs. The bacterial population most likely contained predominantly 11 Gs with a lower proportion of 10 Gs. Proteomic analysis using liquid chromatography tandem mass spectrometry (*12*) found that, in the whole cell of L2228, only 2.3% of the FhaB protein was detected as peptides and derived mainly from the first 350 aa of the FhaB protein. In contrast, in the Fha-positive isolate (L2248), 30.7% of the FhaB protein was detected as peptides and derived from the entire protein. However, in the supernatant of L2228, we detected peptides across the entire FhaB protein and at a higher coverage of 22.7% than for whole-cell FhaB. For the Fha-positive isolate, we detected 52.0% of the FhaB across the entire protein. Western immunoblotting could not detect any FhaB in supernatant or whole-cell proteins of the Fha-negative isolate.

**Figure 2:**
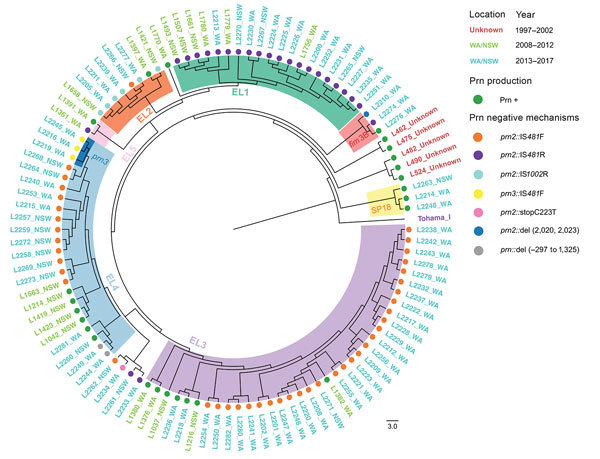
Phylogenomic relationship of 105 *Bordetella pertussis* epidemic isolates from Australia. The minimum-evolution tree was constructed using 705 SNPs. The isolates are labeled by name (L numbers), followed by states with color to indicate years of isolates. Shaded areas inside circle indicate ELs (EL1–EL5) and branches with isolates belonging to SP18 and those carrying *fim*3B and *prn*3 alleles. Prn-positive isolates and different mechanisms of Prn-negative isolates are marked by circles with different colors. Scale bar represents 3 SNPs. EL, epidemic lineage; NSW, New South Wales; Prn, pertactin; SNP, single-nucleotide polymorphism; SP, SNP profile; WA, Western Australia.

Together with the 27 *B. pertussis* isolates from Australia previously sequenced, we analyzed a total of 105 *B. pertussis* isolates to determine their genomic relationships (Figure 2). Five preepidemic SP13 isolates from 1997–2002 were ancestral to the SP13 epidemic clade as expected; 3 noncluster I (*ptxP1*) isolates grouped together as a separate clade outside SNP cluster I. Most (68/75) isolates grouped into 4 previously defined ELs (EL1–EL4) (*4*). However, no isolates from the new epidemic fell into the 2008–2012 EL5. Four isolates (L2233, L2234, L2261, and L2262) did not cluster with any of the ELs. 

Prn-positive isolates from the 2008–2012 and 2013–2017 epidemics were distributed among different lineages. Prn-negative isolates were largely grouped by mechanism of inactivation in different ELs. Prn-negative isolates in EL1 and EL4 were caused by IS*481*R insertion. All but 1 Prn-negative isolate in EL2 was caused by IS*1002* insertion; the exception was an IS*481*R insertion. Prn-negative isolates in EL3 were caused by IS*481*F insertion. Three Prn-negative isolates with new inactivation mechanisms found in Australia were distributed in EL4 (*prn*::del [−297, 1325]; note that the *prn* allele was indeterminate) and non-ELs (*prn2*::stop [C233T]).

EL1 contained isolates from NSW (6/20) and WA (14/20). EL2 was a small lineage (8 isolates), but these isolates were from both periods and both states. EL3 was predominantly a WA lineage; 30/33 isolates from WA and nearly half of the WA isolates (30/61) from 2013–2017 were EL3. EL4 was largely an NSW lineage (14/23 isolates).

## Conclusions

The 2013–2017 pertussis epidemic in Australia was predominantly caused by Prn-negative strains, with local and interstate expansion of 4 epidemic lineages. The ongoing expansion of Prn-negative strains is most likely due to continued vaccine selection pressure because Australia has been using ACVs that contain Prn since their introduction. This observation contrasts with the declining circulation of Prn-negative strains in Japan, where changes in the vaccine probably caused the decrease because 2 of the 3 vaccines used after 2012 did not contain Prn (*13*). The emergence of an Fha-negative and Prn-negative *B. pertussis* in Australia may offer higher potential to escape ACV-induced immunity.

Our results provide further evidence of *B. pertussis* evolution under vaccine selection. Continued surveillance of *B. pertussis* will provide a better understanding of the effect of vaccination on the evolution of the pathogen and optimize strategies to reduce the occurrence of pertussis.

Appendix 1 Strain information of 78 *Bordetella pertussis* isolates, Australia, 2013–2017, and single-nucleotide polymorphisms among these isolates.

Appendix 2 Additional methods for the study of pertactin-negative and filamentous hemagglutinin-negative *Bordetella pertussis*, Australia, 2013–2017.
