# Supplementary material for: Pertactin-Negative and Filamentous Hemagglutinin-Negative Bordetella pertussis, Australia, 2013–2017
Source: Emerg Infect Dis. 2019 Jun;25(6):1196–9. doi: 10.3201/eid2506.180240 (PMC6537726; doi:10.3201/eid2506.180240)
Supplement: Appendix 2 — Additional methods for the study of pertactin-negative and filamentous hemagglutinin-negative Bordetella pertussis, Australia, 2013–2017. [file 18-0240-Techapp-s2.pdf]

Article DOI: https://doi.org/10.3201/eid2505.180240

Pertactin-Negative and Filamentous Hemagglutinin-Negative *Bordetella pertussis*, Australia, 2013–2017

Appendix 2

Materials and Methods

Bacterial Strains and Genomic DNA Preparation

Bordetella pertussis were sampled from Princess Margaret Hospital for Children, Subiaco WA, and Westmead Hospital, Sydney, NSW, from 2013 to 2017. A total of 78 isolates (details of the strains used in this study are given in Appendix 2 Table 1, https://wwwnc.cdc.gov/EID/article/25/6/18-0240-App1.xlsx) were acquired from cryovials stored in −80°C and inoculated on Bordet Gengou agar (Becton Dickinson, Sparks, MD, USA, supplemented with 7% horse blood). All samples were incubated at 37°C for 4–5 days. Genomic DNA were extracted and purified by the phenol-chloroform method.

Fewer isolates were collected in NSW than WA, possibly due to different numbers of cases coming into the collecting hospital and different uptake of direct PCR as the default method of laboratory diagnosis. The number of isolates did not reflect the population size of the state nor the local incidence of pertussis.

Genome Sequencing and Assembly

Whole-genome sequencing (WGS) of isolates was performed by Illumina MiSeq/NextSeq (Illumina, Scoresby, VIC, Australia). DNA libraries were constructed using Nextera XT Sample preparation kit (Illumina Inc., San Diego, CA, USA). The fragment size distribution of the tagmented DNA was analyzed using a High Sensitivity DNA assay kit (Caliper Life Science, Hopkinton, MA, USA). Genome sequencing was done in a multiplex of 60 samples. Libraries were sequenced either using the MiSeq Personal Sequencer (Illumina Inc) or NextSeq sequencer (Illumina Inc.). Raw reads were submitted to GenBank under the BioProject PRJAN432286). De novo assembly was performed for all sequencing data using

SPAdes (version 3.7.0). Sanger sequencing (ABI 3730 Capillary Sequencer, Ramaciotti Center for Genomics, UNSW Sydney, Kengsinton, NSW, Australia) was performed to confirm the genotypes of *prn*, *fim2*, and *fhaB*. *prn* was amplified by PCR and sequenced using the published primers (PRN1157 5′-CACCGCACGGCAATGTCAT-3′, PRNBR 5′-CGGATTCAGGCGCAACTC-3′) (*1*), while newly designed primers (fhaBF 5′-TCAAGTTGGGCACTGGAGAC-3′, fhaBR 5′-CTGGGCTATTTCGACGTGGT-3′) were used for *fhaB* and (fim2F 5′-ACGGCATTGGCAGTGGTGGA-3′, fim2R 5′-CACACAAACTTGATGGGCGA-3′) for *fim2*.

Bioinformatics Analysis

SNP detection used a combination of mapping by Burrow-Wheeler Alignment (BWA) tool (version 0.7.12), SAMtools (version 0.1.19), and alignment by progressiveMauve (version snapshot_2015_02–25). Phylogenetic trees were constructed using MEGA (version 5.2.1), the Minimum Evolution tree was applied based on the Close-Neighbor-Interchange (CNI) method and the bootstrap analysis was based on 1,000 replicates (SNPs files shown in Appendix 2 Table 2). Short insertion/deletions (indels) (<100 bp) were identified by SAMtools.

Western Immunoblotting

Western immunoblotting was used to detect the expression of Pertussis toxin (Ptx), Pertactin (Prn) and Filamentous hemagglutinin (Fha). Bacterial suspensions were mixed with Laemmli buffer (5% β-mercaptoethanol were added before use) and boiled for 10 min. Proteins separated by sodium dodecyl sulfate-polyacrylamide gel electrophoresis (SDS-PAGE) were transferred onto a polyvinylidene difluoride membrane at 100 V for 1.5 hr. Membranes were washed 3 times in washing buffer (50 mM Tris-HCl pH7.6, 150 mM NaCl, 0.05% Tween-20) before incubated, first with blocking buffer (washing buffer, 5% [wt/vol] skim milk powder] at room temperature (RT) for 2 hr, and then with mouse polyclonal antibodies against Ptx, Fha, and Prn (diluted in 1:1,000, 1:1,000, and 1:3,000, respectively with blocking buffer) at 4°C, overnight. After being washed 3 times with washing buffer, membranes were incubated with sheep antimouse (IgG) antibodies conjugated to horse radish peroxidase (Abcam) diluted in 1: 20,000 with blocking buffer at RT for 1 hr and 15 min.

Sample Preparation for LC-MS/MS

Liquid chromatography–mass spectrometry (LC-MS/MS) was performed on 2 samples (L2228 and L2248) to confirm the expression of Fha. Each sample had 3 biological repeats. For typsin digestion, 10 mM Dithiothreitol (DTT) was added to samples (10 μg/sample) for the reduction of disulfide bonds and incubated at 37°C for 30 min. Following incubation, samples were alkylated through the addition of 20 mM Iodoacetamide (IA) and incubated for an additional 30 min at 37°C in the dark. Samples were then incubated at 37°C overnight after Trypsin was added at a ratio of 1:100 enzymes to protein. Strong cationic exchange (SCX) was performed using 200 μL SCX stage tips (Thermo Fisher Scientific) on each sample for clean-up. After SCX, samples were then dried using a Speedivac (Thermo Fisher Scientific) and resuspended in 10 μL of 0.1% formic acid.

LC-MS/MS

Samples were analyzed in the Bioanalytical Mass Spectrometry Facility (BMSF) at the University of New South Wales using LC-MS/MS. Protein identifications were searched against the NCBI database using the Mascot server (v.2.51) (Matrix Science).

Reference

1. Fry NK, Neal S, Harrison TG, Miller E, Matthews R, George RC. Genotypic variation in the *Bordetella pertussis* virulence factors pertactin and pertussis toxin in historical and recent clinical isolates in the United Kingdom. Infect Immun. 2001;69:5520–8. PubMed http://dx.doi.org/10.1128/IAI.69.9.5520-5528.2001
